# Impact of Previous Thoracotomy on Outcomes of Open Thoracoabdominal Aortic Aneurysm Repair: A Retrospective Propensity Score-Matched Analysis

**DOI:** 10.3390/jcm15030963

**Published:** 2026-01-25

**Authors:** Muhyung Heo, Siwon Oh, Suryeun Chung, Dong Seop Jeong, Wook Sung Kim, Yang Hyun Cho, Kiick Sung

**Affiliations:** 1 Department of Thoracic and Cardiovascular Surgery, Samsung Medical Center, Seoul 06351, Republic of Korea; mhyung1016@gmail.com (M.H.); siwon.oh@samsung.com (S.O.); suryeun.chung@samsung.com (S.C.); dongseop.jeong@samsung.com (D.S.J.); wooksung.kim@samsung.com (W.S.K.); 2 Department of Thoracic and Cardiovascular Surgery, Korean Armed Forces Capital Hospital, Seongnam-si 13574, Gyeonggi-do, Republic of Korea

**Keywords:** thoracoabdominal aortic aneurysm, repeat thoracotomy, redo surgery, propensity score matching

## Abstract

**Objectives**: Open repair remains widely used for TAAA (Thoracoabdominal Aortic Aneurysm), but disease progression may require reoperation via repeat thoracotomy, which is technically challenging. These procedures involve increased risks due to adhesions and altered anatomy. This study aims to evaluate the impact of repeat thoracotomy on early surgical outcomes in TAAA patients. **Methods**: We conducted a retrospective cohort study of 214 patients who underwent open TAAA repair between June 1996 and March 2023. Among them, 30 underwent repeat thoracotomy (RT), and 184 underwent their first thoracotomy (FT). To reduce baseline discrepancies, propensity score matching (3:1) was performed, resulting in 22 RT patients matched with 45 FT patients. The primary outcome was 30-day mortality, while secondary outcomes included postoperative complications. **Results**: In the matched cohort, the median operative time was longer in the RT group (500 min, IQR [476.0–552.0]) compared to the FT group (459.0 min, IQR [426.5–514.0]; *p* = 0.037). Thirty-day mortality was similar between groups (RT: 4.5%, FT: 2.2%, *p* = 0.433). No cases of paraplegia occurred. Postoperative bleeding was observed more frequently in the RT group (RT: 13.6% vs. FT: 2.2%, *p* = 0.050), suggesting a potential difference, but statistical significance was not reached. Other major complications showed no significant differences. **Conclusions**: In this propensity score matched analysis, repeat thoracotomy was not associated with statistically significant differences in early outcomes following open TAAA repair. These findings should be interpreted cautiously and suggest that prior left thoracotomy may not be associated with worse early outcomes in experienced centers.

## 1. Introduction

Managing thoracoabdominal aortic aneurysms (TAAA) requires an individualized approach tailored to the patient’s condition and the extent of intervention. Open repair remains a well-established modality, with contemporary series reporting acceptable operative mortality, low rates of major complications, and durable outcomes in high-volume and experienced centers [1,2,3]. Despite these positive results, late surgical failures remain challenging, often necessitating redo procedures due to disease progression in the residual aorta after prior descending aorta surgery [4]. Approximately 15% to 25% of patients undergoing TAAA open repair have a history of prior descending thoracic aorta surgery, indicating that reoperative scenarios are frequently encountered in clinical practice [3,5]. Redo surgeries present distinct technical challenges and heightened risks, particularly when repeat thoracotomy is required. Previous surgery involving the descending thoracic aorta complicates subsequent procedures due to adhesions and the complex dissection needed to address intricate lesions [6]. As a result, repeat thoracotomies require more meticulous planning than initial operations.

Predisposing factors such as connective tissue disorders, aortic dissection, and Crawford classification types II or III are often observed in patients undergoing redo TAAA procedures procedures [5,7,8]. However, prior studies have largely focused on reporting outcomes after reoperative thoracic aortic surgery, often including both descending thoracic and thoracoabdominal aneurysm repair, without specifically evaluating whether repeat thoracotomy itself contributes to operative risk in redo TAAA repair [5,8]. To address this gap, our study analyzes outcomes of open TAAA repair by comparing cases involving repeat thoracotomy with first-time TAAA surgeries using propensity score matching to achieve a balanced comparison. Specifically, we aimed to evaluate our institutional experience with TAAA open repair and assess the impact of repeat thoracotomy on early outcomes.

## 2. Methods

### 2.1. Patients

This retrospective cohort study included 214 patients who underwent TAAA surgery at Samsung Medical Center from June 1996 to March 2023. Among these patients, 30 required repeat thoracotomy due to prior descending aorta surgery, comprising the Repeat Thoracotomy (RT) group. Patients without a history of descending thoracic aorta surgery who underwent their first thoracotomy for thoracoabdominal repair were designated as the First Thoracotomy (FT) group. To reduce baseline discrepancies, propensity score matching at a 3:1 ratio was conducted based on age, replacement level, comorbidities, aneurysm type, and other relevant baseline characteristics (Table 1). After matching, the RT group included 22 patients, and the FT group included 45. This study was approved by the Institutional Review Board of Samsung Medical Center (SMC 2024-09-036-001, approved 15 October 2024), with informed consent waived as all data were de-identified.

### 2.2. Definitions

Thoracoabdominal aortic aneurysms were classified by the Crawford classification. For patients in the RT group, classification was based on the extent of reoperation and replacement level, employing the Crawford classification for consistency. The primary outcome was 30-day mortality. Respiratory complications were defined as the requirement for mechanical ventilation beyond 48 h. Early complications were defined as adverse events occurring within 30 days postoperatively, including permanent spinal cord injury resulting in paraplegia, acute renal failure necessitating dialysis, major ischemic or hemorrhagic stroke, and the need for extracorporeal life support.

### 2.3. Surgical Procedure

Anesthesia was induced with continuous pressure monitoring via a right radial arterial line, and one-lung ventilation was achieved using a double-lumen endotracheal tube. Additional monitoring included a second arterial line in the right femoral artery and a Swan-Ganz catheter inserted through the right internal jugular vein. Patients were positioned semi-laterally for a thoracoabdominal incision, which was extended to the abdominal midline. Partial cardiopulmonary bypass was initiated using cannulas in the left femoral artery and vein. Selective perfusion of the celiac, renal, and superior mesenteric arteries was employed to minimize organ ischemia. To prevent spinal cord ischemia in replacements extending below T10, a lumbar drain was used to maintain cerebrospinal fluid (CSF) pressure below 10 mmHg during surgery. Motor-Evoked Potentials (MEP) and Somatosensory-Evoked Potentials (SSEP) monitoring began after induction and before positioning in non-emergency cases, along with CSF drainage for patients at higher risk of spinal cord ischemia. In response to signal changes, continuous neuromonitoring facilitated immediate corrective interventions, such as adjusting perfusion flow or reducing cross-clamp time.

### 2.4. Statistical Analysis

Statistical analyses were conducted using R Statistical Software (version 4.2.0; R Foundation for Statistical Computing, Vienna, Austria). Continuous variables were expressed as mean (standard deviation) or median (interquartile range), while categorical variables were reported as proportions. Group comparisons were performed using the Chi-square or Fisher’s Exact test for categorical variables and the student *t*-test or the Mann-Whitney U test for continuous variables. The propensity score–matched analysis was designed to enable a more balanced and rigorous comparison of population-averaged outcomes between patients with and without repeat thoracotomy. To preserve the number of patients available for analysis while maintaining adequate balance, a 3:1 matching ratio was selected. Propensity score matching was performed using the nearest neighbor method with a caliper width of 0.2. Due to the small sample size of the RT group, not all baseline characteristics variables from Table 1 were used for matching. Instead, six variables were selected based on the comparison of baseline characteristics between the RT and FT groups, with a *p*-value ≤ 0.2. These variables included age, sex, connective tissue disease, chronic aortic dissection, cardiac surgery history, and Crawford type. After matching, the balance of variables was assessed using standardized mean differences (SMD), confirming that all SMD values were ≤0.2 (Supplementary Figure S1).

For comparisons between the matched groups, categorical and continuous variables were analyzed using the generalized estimating equation (GEE). Variables exhibiting complete separation were excluded from the GEE models to ensure model stability. Outcome analysis was conducted on the overall cohort using multivariable logistic regression for categorical variables and multiple linear regression for continuous variables. Each outcome was adjusted for covariates with a *p*-value ≤ 0.2 through backward selection, along with adjustment for the presence of repeat thoracotomy. Statistical significance was defined as a *p*-value < 0.05.

## 3. Results

### 3.1. Demographics and Baseline Characteristics

In the overall cohort, the median age was significantly lower in the RT group (49.1 years, IQR [40.1–56.3]) compared to the FT group (60.2 years, IQR [43.8–69.9]; *p* = 0.019) (Table 1). The RT group also demonstrated a higher prevalence of connective tissue disease (50.0% vs. 29.9%, *p* = 0.049) and chronic aortic dissection (83.3% vs. 62.0%, *p* = 0.039). Moreover, Crawford type III aneurysms were more frequent in the RT group than in the FT group (Table 1). Following propensity score matching, the median age in the RT group was 53.0 years (IQR [47.2–68.3]) compared to 53.3 (IQR [42.5–68.0]) years in the FT group, with no statistically significant difference (*p* = 0.793). Additionally, there were no significant differences between the matched groups regarding gender, prevalence of connective tissue disease, chronic dissection, or other key clinical variables.

**Figure 1:**
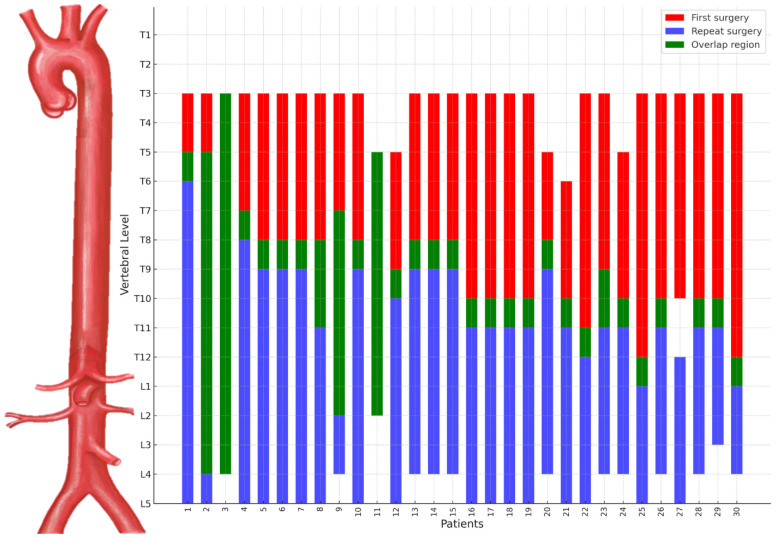
This figure shows the range of aortic replacement levels in repeat thoracotomy patients. Blue bars represent the first surgery, red bars the repeat surgery, and green-highlighted regions indicate overlap in replacement levels. Repeat surgeries typically extend below the initial site, though some cover the entire first surgery range, indicating full re-replacement. This distribution provides anatomical context regarding the relationship between initial descending thoracic aortic repair and subsequent thoracoabdominal extension during repeat thoracotomy.

Table 2 presents a detailed description of the RT group. Most patients had previously undergone descending thoracic aorta replacement (86.7%), with aneurysms being the main indication for reoperation (86.6%). The median interval between the initial and repeat surgeries was 3.4 years (IQR [1.0–4.9]). As shown in Figure 1, repeat thoracotomies frequently required a lower thoracic approach compared to the initial surgical intervention, reflecting extension of prior descending thoracic aortic repair into the thoracoabdominal segment.

### 3.2. Operative Data and Postoperative Outcomes

Operative data revealed no significant differences between the RT and FT groups in the overall cohort (Table 3). However, in the propensity-matched cohort, the median operation time was significantly longer in the RT group (500.0 min, IQR [476.0–552.0]) than the FT group (459.0 min, IQR [426.0–514.0]; *p* = 0.037). There were no significant differences between the groups in terms of cardiopulmonary bypass (CPB) time, aortic cross-clamp (ACC) time, use of lumbar drains, or MEP/SSEP monitoring (Table 3).

The 30-day mortality rates were comparable between the RT group (3.3%) and the FT group (5.4%) in the overall cohort (OR = 0.626, 95% CI: 0.033–3.653, *p* = 0.666). In the propensity-matched cohort, the 30-day mortality rates were 4.5% and 2.2% for the RT and FT groups, respectively (OR = 3.095, 95% CI: 0.184–52.158, *p* = 0.432) (Table 4). Importantly, no cases of paraplegia were reported in either group within the matched cohort. Acute renal failure requiring dialysis occurred in 13.6% of RT patients and 11.1% of FT patients (*p* = 0.799). Major stroke occurred in 9.1% of patients in the RT group but was not observed in the FT group. Postoperative bleeding was observed in 13.6% of the RT group and 2.2% of the FT group, with a higher proportion in the RT group. However, this difference was not statistically significant (OR = 10.263, 95% CI: 0.997–105.621, *p* = 0.050). Respiratory complications, hospital stay, and ICU duration showed no statistically significant differences between the two groups. Other complications, including gastrointestinal complications, graft infection, and ECLS, could not be analyzed due to data limitations.

## 4. Discussion

This study evaluated the impact of repeat thoracotomy on early surgical outcomes in patients undergoing open thoracoabdominal aortic aneurysm (TAAA) repair with prior left thoracotomy. When the overall cohort was stratified into first-time thoracotomy (FT) and repeat thoracotomy (RT) groups, differences in baseline disease characteristics were observed, particularly with respect to connective tissue disease and chronic aortic dissection.

Consistent with prior studies [5,8], the RT group demonstrated a higher prevalence of connective tissue disorders, reflecting the progressive nature of aortic disease in this population. Notably, chronic aortic dissection was highly prevalent in the RT group, accounting for approximately 80% of cases, and remained frequent in the overall cohort (approximately 60%). These proportions are higher than those reported in several prior series, in which chronic aortic dissection has typically accounted for about 30–40% of patients undergoing open TAAA repair [5,8]. Similarly, the prevalence of connective tissue disease in our cohort was relatively high, affecting approximately 30% of patients overall and nearly 50% of those in the RT group, compared with rates of roughly 10–15% reported in previous studies [5,8].

Many patients in the RT group had previously undergone descending thoracic aorta replacement, which influenced the extent of replacement required during subsequent TAAA surgery. Taken together with the baseline disease profile of this cohort, these findings suggest that repeat thoracotomy was commonly required due to progressive aortic disease extending beyond the initially treated segment, rather than isolated technical failure of the prior repair.

Given the inherent clinical differences between patients undergoing first-time and repeat thoracotomy, propensity score matching was applied to facilitate a more balanced comparison of early outcomes between groups. Despite the increased technical complexity and extended operative times associated with repeat thoracotomy during open TAAA repair, our results demonstrated no significant differences in early adverse outcomes, including mortality and major complications, compared to first-time TAAA surgeries. These findings underscore that, with meticulous preoperative planning and precise intraoperative execution, open TAAA repair performed in the setting of repeat thoracotomy can achieve early outcomes without a clear increase in adverse events compared with first-time TAAA procedures. 

### 4.1. Challenges and Technical Considerations of Repeat Thoracotomy

Repeat thoracotomy presents distinct challenges, primarily due to adhesions, scarring, and altered anatomy from previous surgeries. These factors complicate surgical dissection, elevate the risk of bleeding and injury to surrounding structures, and contribute to prolonged operative times, ultimately increasing surgical risk and complexity. Our data confirmed these anticipated challenges, as indicated by the significantly longer operative times observed in the RT group compared to the FT group (500.0 min vs. 459.0 min, *p* = 0.037). This finding aligns with previous studies highlighting the increased technical demands of redo aortic surgery [9,10]. Despite these challenges, our analysis found no significant increase in early mortality or major complications, such as renal failure requiring dialysis, stroke, or paraplegia, in the RT group compared to the FT group. The absence of significant differences in these critical outcomes suggests that advanced surgical techniques and careful intraoperative management can mitigate the inherent risks of repeat thoracotomy. However, postoperative bleeding was observed at a higher rate in the RT group compared to the FT group (13.6% vs. 2.2%, *p* = 0.050). Although not statistically significant, the possibility of bleeding during open TAAA repair in the setting of repeat thoracotomy should not be overlooked.

Although surgical site infection was not specifically evaluated as an outcome in this study and no clear signal was identified, repeat thoracotomy inherently involves extensive dissection and prolonged operative time. Accordingly, while no conclusions can be drawn from the present data, careful attention to perioperative infection prevention remains warranted in this clinical setting.

### 4.2. Intraoperative Strategies to Minimize Risks

Several intraoperative strategies likely contributed to the favorable outcomes observed in the RT group. During aortic cross-clamping, selective perfusion techniques for the celiac, renal, and superior mesenteric arteries were crucial in minimizing organ ischemia. Additionally, CSF drainage was routinely employed when the replacement extended below the T10 level to mitigate the risk of spinal cord ischemia, a well-known complication of thoracoabdominal aortic surgery. MEP and SSEP monitoring, in conjunction with CSF drainage, enabled real-time assessment of spinal cord perfusion and immediate corrective measures when signal changes were detected. These approaches are well-documented in the literature as effective methods for reducing the incidence of paraplegia and other neurological complications in complex aortic surgeries [11,12,13,14]. Especially in the RT group, there were no cases of paraplegia, which can be attributed to the protective strategies described above and the concept of staged operations. Most repeat surgeries involved replacement below the segment treated in the initial surgery, similar to the staged TAAA approach, which has been shown to reduce the risk of paraplegia [15,16]. Studies indicate that following the initial surgery, remodeling of collateral vessels in large muscles provides blood flow to the spinal cord, helping maintain perfusion after descending aortic aneurysm repair. Likewise, the interval between the initial and redo surgeries may allow for developing a more robust collateral blood supply, potentially reducing the risk of ischemia.

### 4.3. Comparative Outcomes

Early studies have reported outcomes of open thoracoabdominal aortic aneurysm (TAAA) repair in patients with prior thoracic or aortic surgery; however, the specific contribution of repeat left-sided thoracotomy itself has been less well characterized [10,17]. Among the earliest reports focusing on this issue, Kawaharada et al. described open TAAA repair through redo left-sided thoracotomy following prior descending thoracic aortic surgery, reporting no statistically significant differences in early complications despite prolonged operative times related to adhesions and altered anatomy [9]. The propensity-matched analysis revealed no statistically significant differences in early mortality between the RT and FT groups, indicating repeat thoracotomy was not associated with worse early outcomes, rather than demonstrating equivalence. This finding is particularly significant given that nearly 90% of redo surgeries in the RT group were necessitated by disease progression in the residual aorta—a scenario frequently encountered in patients with connective tissue disorders or chronic aortic dissection

In our study, early mortality within 30 days was observed in 11 of 214 patients, with only one death (3.3%) in the redo group. Bleeding was the most common cause of mortality, accounting for 45.5% (5 cases) of deaths (Supplementary Table S1). Among these, one bleeding-related death in the RT group occurred during emergency surgery for impending rupture of a native aortic aneurysm at a level not previously operated on. In this case, lung parenchymal injury during the procedure resulted in severe bleeding and eventual death. Although rare, the bleeding-related death observed in the RT group highlights the need for heightened caution when performing open TAAA repair in the setting of repeat thoracotomy, particularly in emergency settings. Although this difference did not reach conventional statistical significance, the magnitude of the observed effect suggests that postoperative bleeding may represent a clinically relevant and hypothesis-generating trade-off associated with repeat thoracotomy.

Taken together, these findings suggest that prior left thoracotomy may not be associated with worse early outcomes in patients undergoing open TAAA repair at experienced centers. However, clinically anticipated risks related to repeat thoracotomy, particularly bleeding associated with adhesions and lung injury, should be carefully considered during surgical planning and perioperative management.

### 4.4. Limitations

This study has several limitations. First, it is a retrospective, single-center study, which may limit the generalizability of our findings. Moreover, as the study reflects outcomes from an experienced center with established perioperative protocols, the results may not be directly generalizable to other institutional settings with different levels of expertise or infrastructure. Although propensity score matching was employed to balance baseline characteristics, residual confounding cannot be excluded. In addition, alternative analytical approaches may yield different estimates, and residual confounding related to methodological constraints cannot be fully excluded. In particular, unmeasured factors such as prior operative complexity, emergency status, renal function or other clinical characteristics may have influenced both the likelihood of repeat thoracotomy and early outcomes. Furthermore, the small sample size of the data may limit the statistical interpretation of the results, highlighting the need for future analyses with larger datasets. Lastly, while this study focused on early outcomes, the long-term impact of repeat thoracotomy on survival and quality of life remains unclear and warrants further investigation.

## 5. Conclusions

In conclusion, in this propensity score–matched analysis, no statistically significant differences in early clinical outcomes between FT and RT groups undergoing open TAAA repair. These findings suggest that prior left thoracotomy, when present in patients undergoing open TAAA repair, may not be associated with worse early outcomes in appropriately selected patients treated at experienced centers. However, given the limited sample size and the potential for residual confounding, these results should be interpreted with caution. Rather than serving as definitive evidence to guide clinical decision-making, our findings underscore the importance of individualized assessment and institutional expertise when considering open TAAA repair in patients with a history of prior left thoracotomy for descending thoracic or thoracoabdominal aortic surgery. Further studies with larger cohorts and longer follow-up are warranted to better define patient selection and long-term outcomes in this high-risk population.

## Figures and Tables

**Table 1:** Baseline clinical characteristics.

Variable	Overall Cohort				Propensity-Matched Cohort			
	Overall (*n* = 214)	FT Group (*n* = 184)	RT Group (*n* = 30)	*p* Value	Overall (*n* = 67)	FT Group (*n* = 45)	RT Group (*n* = 22)	*p* Value
Age (years)	57.3 (42.8–69.2)	60.2 (43.8–69.9)	49.1 (40.1–56.3)	0.019	53.1 (42.8–68.2)	53.3 (42.5–68.0)	53.0 (47.2–68.3)	0.793
Male	152 (71.0)	127 (69.0)	25 (83.3)	0.166	46 (68.7)	29 (64.4)	17 (77.3)	0.365
Connective tissue disease	70 (32.7)	55 (29.9)	15 (50.0)	0.049	29 (43.3)	21 (46.7)	8 (36.4)	0.182
Marfan’s syndrome	61 (28.5)	49 (26.6)	12 (40.0)	0.198	25 (37.3)	18 (40.0)	7 (31.8)	0.189
Loeys Dietz syndrome	9 (4.2)	6 (3.3)	3 (10.0)	0.116	4 (6.0)	3 (6.7)	1 (4.5)	>0.999
Chronic Aortic dissection	139 (65.0)	114 (62.0)	25 (83.3)	0.039	50 (74.6)	33 (73.3)	17 (77.3)	0.652
Crawford type				<0.001				0.786
I	15 (7.0)	14 (7.6)	1 (3.3)		3 (4.5)	2 (4.4)	1 (4.5)	
II	90 (42.1)	87 (47.3)	3 (10.0)		11 (16.4)	9 (20.0)	2 (9.1)	
III	66 (30.8)	58 (47.3)	23 (76.7)		43 (64.2)	27 (60.0)	16 (72.7)	
IV	28 (13.1)	25 (13.6)	3 (10.0)		10 (14.9)	7 (15.6)	3 (13.6)	
Hypertension	135 (63.1)	113 (61.4)	22 (73.3)	0.293	45 (67.2)	27 (60.0)	18 (81.8)	0.093
Diabetes	15 (7.0)	13 (7.1)	2 (6.7)	>0.999	4 (6.0)	2 (4.4)	2 (9.1)	0.263
COPD	5 (2.3)	4 (2.2)	1 (3.3)	0.534	2 (3.0)	1 (2.2)	1 (4.5)	>0.999
Smoking history	91 (42.9)	76 (41.8)	15(50.0)	0.518	35 (52.2)	21 (46.7)	14 (63.6)	0.110
Chronic kidney disease *	13 (6.1)	11 (6.0)	2 (6.7)	>0.999	2 (3.0)	0	2 (9.1)	-
Peripheral artery disease	16 (7.5)	13 (7.1)	3 (10.0)	0.476	7 (10.4)	4 (8.9)	3 (13.6)	0.433
History of CVA	16 (7.5)	15 (8.2)	1 (3.3)	0.706	2 (3.0)	1 (2.2)	1 (4.5)	0.433
Previous AAA open repair	20 (9.3)	18 (9.8)	2 (6.7)	0.746	9 (13.4)	8 (17.8)	1 (4.5)	0.215
Previous TEVAR	10 (4.7)	8 (4.3)	2 (6.7)	0.635	4 (6.0)	3 (6.7)	1 (4.5)	0.893
Previous EVAR	2 (0.9)	2 (1.1)	0	>0.999	0	0	0	-
Previous cardiac surgery	94 (43.9)	75 (40.8)	19 (63.3)	0.035	40 (59.7)	29 (64.4)	11 (50.0)	0.126

Data presented as no. (%) or median (interquartile range). * estimated glomerular filtration rate (eGFR) < 60 mL/min/1.73 m^2^. COPD: chronic obstructive pulmonary disease; CVA: cerebrovascular accident; AAA: Abdominal aortic aneurysm; TEVAR: Thoracic endovascular aortic repair; EVAR: Endovascular aneurysm repair.

**Table 2:** RT group previous surgery information.

Variable	Number (*n* = 30)
Type of previous aortic surgery	
Descending thoracic aorta replacement	26 (86.7)
Thoracoabdominal aorta replacement	3 (10.0)
Descending thoracic aorta wrapping	1 (3.3)
Time interval to Repeat surgery (year)	3.4 (1.0–4.9)
Indication of Repeat aortic surgery	
Aneurysm	26 (86.6)
Aneurysm impending rupture	1 (3.3)
Anastomosis site pseudoaneurysm	2 (6.7)
Graft infection	1 (3.3)
Previous surgical approach	
4th ICS	4 (16.0)
5th ICS	13 (52.0)
6th ICS	5 (20.0)
7th ICS	3 (12.0)
Repeat surgical approach	
6th ICS	4 (13.3)
7th ICS	22 (73.3)
8th ICS	4 (13.3)

Data presented as no. (%) or median (interquartile range). ICS: Intercostal space.

**Table 3:** Operative data in the overall and propensity score–matched cohorts.

Variable	Overall Cohort				Propensity-Matched Cohort			
	Overall (*n* = 214)	FT Group (*n* = 184)	RT Group (*n* = 30)	*p* Value	Overall (*n* = 67)	FT Group (*n* = 45)	RT Group (*n* = 22)	*p* Value
Emergency operation	16 (7.5)	13 (7.1)	3 (10.0)	0.476	2 (3.0)	0	2 (9.1)	-
Operation time(min)	497.0 (426.0–554.0)	492.5 (421.0–554.0)	500.5 (476.0–559.0)	0.369	484.0 (434.5–530.0)	459.0 (426.0–514.0)	500.0 (476.0–552.0)	0.037
CPB time (min)	169.5 (128.0–207.0)	171.0 (125.5–213.0)	168.0 (128.0–192.0)	0.618	168.3 ± 47.1	168.3 ± 46.8	165.2 ± 48.8	0.668
ACC time(min)	157.0 (118.0–180.0)	157.0 (110.0–184.0)	159.0 (128.0–174.0)	0.945	159.0 (135.0–174.0)	159.0 (138.5–180.0)	155.0 (131.0–168.0)	0.170
Rectal temperature (°C)	30.3 (29.6–31.4)	30.4 (29.6–31.5)	30.1 (29.4–30.7)	0.051	30.1 (29.5–30.8)	30.1 (29.5–30.9)	30.1 (29.4–30.7)	0.123
Lumbar drainage	191 (89.3)	162 (88)	29 (96.7)	0.213	61 (91.0)	40 (88.9)	19 (86.4)	0.150
MEP and SSEP monitoring	159 (74.3)	132 (71.2)	27 (90.0)	0.058	54 (80.6)	35 (77.8)	19 (86.4)	0.171

Data presented as no. (%), median (interquartile range) or mean ± standard deviation. CPB: Cardiopulmonary bypass; ACC: Aortic cross clamp; MEP: Motor-Evoked Potentials; SSEP: Somatosensory-Evoked Potentials.

**Table 4:** Post-operative outcomes in the overall and propensity score–matched cohorts.

Variable	Overall Cohort ^a^						Propensity-Matched Cohort					
	Overall (*n* = 214)	FT Group (*n* = 184)	RT Group (*n* = 30)	OR/Coef	95% CI	*p* Value	Overall (*n* = 67)	FT Group (*n* = 45)	RT Group (*n* = 22)	OR/Coef	95% CI	*p* Value
30-day mortality ^b^	11 (5.1)	10 (5.4)	1 (3.3)	0.626	0.033–3.653	0.666	2 (3.0)	1 (2.2)	1 (4.5)	3.095	0.184–52.158	0.433
Paraplegia ^b^	8 (3.7)	8 (4.3)	0 (0.0)			-	0	0	0			-
Dialysis ^b^	23 (10.7)	20 (10.9)	3 (10.0)	3.421	0.642–15.362	0.117	8 (11.9)	5 (11.1)	3 (13.6)	1.232	0.247–6.135	0.799
major stroke ^b^	13 (6.1)	10 (5.4)	3 (10.0)	2.872	0.579–11.480	0.152	2 (3.0)	0	2 (9.1)			-
Respiratory complication ^b^	39 (18.2)	36 (19.6)	3 (10.0)	1.315	0.272–4.891	0.701	9 (13.4)	6 (13.3)	3 (13.6)	1.068	0.222–5.149	0.935
Postoperative bleeding ^b^	19 (8.9)	16 (8.7)	3 (10.0)	4.765	0.859–23.927	0.057	4 (6.0)	1 (2.2)	3 (13.6)	10.263	0.997–105.621	0.050
Gastrointestinal complication ^b^	5 (2.3)	4 (2.2)	1 (3.3)	5.12	0.123–81.722	0.247	1 (1.5)	0	1 (4.5)			-
Graft infection ^b^	1 (0.5)	0 (0.0)	1 (3.3)			-	1 (1.5)	0	1 (4.5)			-
ECLS ^b^	3 (1.4)	3 (1.6)	0 (0.0)			-	1 (1.5)	1 (2.2)	0			-
length of stay ^c^	14.5 (10.0–21.0)	15.0 (10.0–21.5)	11.0 (9.0–20.0)	4.209	−10.041–18.459	0.561	14.0 (10.0–20.5)	14.0 (10.0–20.0)	12.5 (10.0–23.0)	−0.273	0.406–1.426	0.394
ICU duration ^c^	3.0 (2.0–5.0)	3.0 (2.0–5.0)	3.0 (2.0–4.0)	3.210	−8.138–14.558	0.578	3.0 (2.0–4.0)	3.0 (2.0–4.0)	3.0 (2.0–4.0)	−0.214	0.477–1.368	0.314

Data presented as no. (%) or median (interquartile range). OR: Odds ratio; Coef: Coefficient; CI: confidence interval; ECLS: Extracorporeal life support; ICU: intensive care unit. ^a^ Adjusting for Age, Sex, Connective tissue disease, chronic aortic dissection, Crawford type, previous cardiac surgery. ^b^ Denoted by OR due to binary outcome. ^c^ Denoted by Coef due to continuous outcome.

## Data Availability

The data presented in this study are available on reasonable request from the corresponding author. The data are not publicly available due to ethical and privacy restrictions.

## Supplementary Materials

The following supporting information can be downloaded at: https://www.mdpi.com/article/10.3390/jcm15030963/s1, Figure S1: Standardized mean difference; Table S1: Features of mortality cases.

## Abbreviations

TAAA	Thoracoabdominal Aortic Aneurysm
RT	Repeat Thoracotomy
FT	First-Time Thoracotomy
MEP	Motor-Evoked Potentials
SSEP	Somatosensory-Evoked Potentials
