# Supplementary material for: Impact of Previous Thoracotomy on Outcomes of Open Thoracoabdominal Aortic Aneurysm Repair: A Retrospective Propensity Score-Matched Analysis
Source: J Clin Med. 2026 Jan 25;15(3):963. doi: 10.3390/jcm15030963 (PMC12898820; doi:10.3390/jcm15030963)
Supplement: Supplementary file 1 [file jcm-15-00963-s001.zip › jcm-4098863-supplementary/Supplementary materials (REV.).pdf]

SUPPLEMENTAL MATERIALS

FIGURE S1. Standardized mean difference. This figure shows the standardized mean difference (SMD) for the 6 covariates used in propensity score matching, including age, sex, connective tissue disease, chronic aortic dissection, Crawford type, and cardiac surgery history. Propensity scores were represented by distance. All covariates had no missing values. SMD was calculated to assess the balance of variables before and after matching, confirming improved balance post-matching.

* Continuous variable.

TABLE S1. Features of Mortality Cases

Cause of death	Overall cohort			Propensity-matched cohort		
	Overall (n = 11)	FT group (n = 10)	RT group (n = 1)	Overall (n = 2)	FT group (n = 1)	RT group (n = 1)
Bleeding	5	4	1	1	0	1
Cardiac cause	2	2	0	0	0	0
Hepatic failure	2	2	0	0	0	0
Multi-organ failure	1	1	0	1	1	0
Graft infection	1	1	0	0	0	0
