# Supplementary figures and images for: Impact of Previous Thoracotomy on Outcomes of Open Thoracoabdominal Aortic Aneurysm Repair: A Retrospective Propensity Score-Matched Analysis
Source: J Clin Med. 2026 Jan 25;15(3):963. doi: 10.3390/jcm15030963 (PMC12898820; doi:10.3390/jcm15030963)

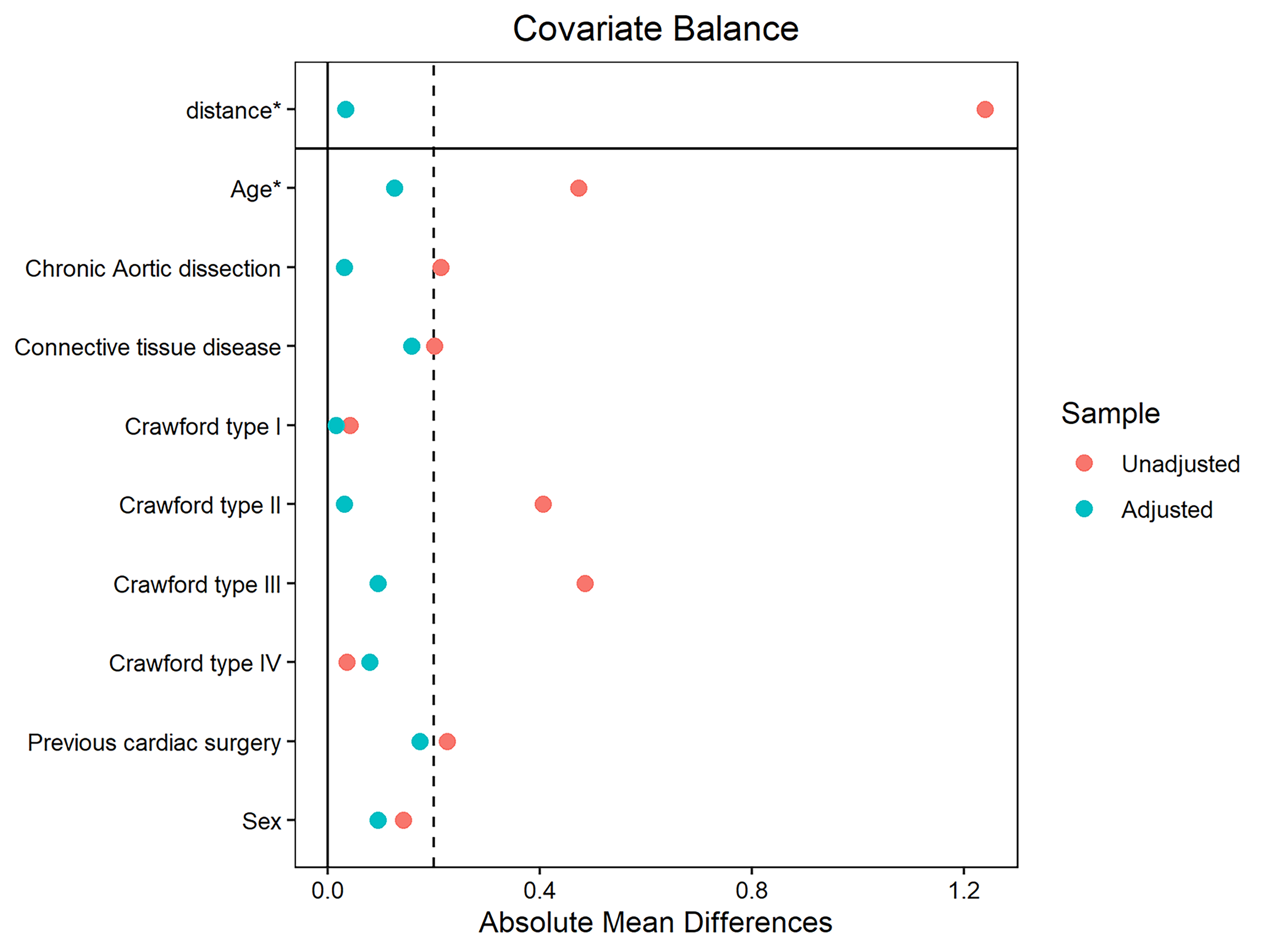

Supplement: Supplementary file 1 [file jcm-15-00963-s001.zip › jcm-4098863-supplementary/Figure S1.tif]
